# Vaginal-perianal or vaginal-perineal compared with vaginal-rectal culture-based screening for Group B Streptococci (GBS) colonization during the third trimester of pregnancy: a systematic review and meta-analysis

**DOI:** 10.1186/s12884-022-04546-w

**Published:** 2022-03-14

**Authors:** Hugh C. G. Nadeau, Courtney Bisson, Xi Chen, Yan D. Zhao, Marvin Williams, Rodney K. Edwards

**Affiliations:** 1 Section of Maternal-Fetal Medicine, Department of Obstetrics and Gynecology, University of Oklahoma Health Sciences Center, 800 Stanton L. Young Blvd, Suite 2400, Oklahoma City, OK 73104 USA; 2 Department of Biostatistics and Epidemiology, University of Oklahoma Health Sciences Center Oklahoma City, Oklahoma City, OK USA

**Keywords:** Pregnancy, Group B Streptococcus, Culture-based Screening, Prenatal care

## Abstract

**Background:**

Screening for maternal anogenital Group B streptococci (GBS) colonization in pregnancy with initiation of intravenous intrapartum antibiotic prophylaxis as indicated has led to a significant reduction in the incidence of neonatal GBS infection. This study aims to evaluate the agreement between vaginal-perianal or vaginal-perineal culture and the more typically used vaginal-rectal culture for screening for maternal anogenital GBS colonization in the third trimester of pregnancy.

**Methods:**

Eligible English-language studies published until January 2020 were retrieved from Scopus, Web of Science, PubMed, Embase, and ClinicalTrials.gov databases. Studies were compiled that assessed for GBS colonization utilizing vaginal-perianal or vaginal-perineal culture and vaginal-rectal culture during the third trimester of pregnancy. Nonoriginal research articles and studies that did not assess pregnant patients, did not use culture-based screening, or did not compare vaginal-perianal or vaginal-perineal culture with vaginal-rectal culture were excluded. The search identified 559 articles with three prospective cohort studies that met inclusion criteria, including 643 participants. Quality was assessed using the Newcastle–Ottawa Scale, and risk of bias was assessed using the Risk of Bias In Non-randomized Studies of Interventions (ROBINS-I) tool. Patient characteristics and associated pain with specimen collection were abstracted. Meta-analyses of both the raw agreement and the Cohen’s kappa statistic were performed.

**Results:**

Within the three included studies, the range of GBS detection was 17.6–34.0%, consistent with the anticipated prevalence of GBS colonization reported in earlier publications. For both raw agreement and Cohen’s kappa coefficient, the test for heterogeneity was not significant, indicating low heterogeneity among studies. The pooled estimate of the raw agreement was 0.97 (95%CI 0.95–0.98) and of the Cohen's kappa coefficient was 0.91 (95% CI: 0.87–0.95), indicating (according to the Landis and Koch criteria) an “almost perfect” agreement between the compared clinical tests. In the two studies that assessed procedure-related patient discomfort, vaginal-rectal swabbing caused more discomfort.

**Conclusion:**

Use of vaginal-perineal culture for assessment of maternal GBS colonization is comparable to the more typically utilized vaginal-rectal culture and is associated with less discomfort.

## Background

Consensus guidelines from the Centers for Disease and Prevention (CDC), American College of Obstetricians and Gynecologists (ACOG) and American Academy of Pediatrics (AAP) have recommended since 1996 intravenous intrapartum antibiotic prophylaxis to reduce the incidence of neonatal infection with *Streptococcus agalactiae* (group B streptococcus; GBS) in the first week of life (early-onset disease, EOD) [1]. While GBS primarily colonizes the gastrointestinal tract, the perianal skin, perineum, and vagina are likely secondarily colonized given proximity to the rectum [2]. When using selective broth media, this bacterium can be isolated by culture from the vagina and rectum of 15–35% of pregnant women [3, 4]. Because doing so results in lower rates of GBS EOD [5], use of a screening-based, as opposed to a risk-factor-based, strategy along with antibiotic prophylaxis for carriers has been recommended in the CDC guideline since 2002 and still is recommended in the current ACOG guideline (Responsibility for maintenance of guidelines for prevention of GBS EOD in newborns has transitioned from the CDC, for the obstetric and pediatric management strategies respectively, to ACOG and AAP) [6].

According to these guidelines, the administration of intrapartum antibiotic prophylaxis has resulted in an 80% reduction in GBS EOD since the early 1990s. There currently are approximately 930 cases per year in the United States, consistent with a rate of 0.25 per 1000 live births [7, 8].

The specimen collection technique suggested by all iterations of the guidelines published in the United States since 1996 involves swabbing both the lower third of the vagina and the rectum [1, 6]. This method is based on a study published in 1977 that found that rectal cultures were positive more often than vaginal cultures [9]. Since then, at least one study has described that most women, almost three-quarters of them, report at least mild pain associated with obtaining the rectal screening sample [10]. Those authors reported no significant difference in GBS recovery when sampling from the rectum compared to the perianal skin and less pain associated with perianal sampling, but they did not evaluate vaginal combined with rectal sampling. However, at least two studies have reported that combined vaginal and perianal sampling yields similar results to combined vaginal and rectal sampling [11, 12].

Therefore, despite the recommendation that GBS screening cultures during pregnancy be collected from the lower vagina and rectum, there is no clear consensus that this approach is superior to vaginal and perianal collection, and the combined vaginal and rectal collection technique may be associated with more, and potentially unnecessary, patient discomfort.

In this review, we aimed to systematically identify, appraise, and summarize the existing data from prospective studies that compare the recovery of GBS in screening cultures of pregnant women when samples are obtained from the combined sites of the vagina and rectum compared to the combined sites of the vagina and perianal skin or the vagina and the perineum. Secondarily, we sought to evaluate the existing evidence relating to pain associated with these alternative culture specimen sampling techniques.

## Methods

### Sources

The study protocol was developed, and a systematic review of the relevant literature was completed, according to the PRISMA (Preferred Reporting Items for Systematic Reviews and Meta-Analyses) statement. The study was registered with PROSPERO (registration number CRD42020163650). The primary objective was to identify studies comparing vaginal-perianal or vaginal-perineal to vaginal-rectal culture for screening for maternal anogenital GBS colonization in the third trimester of pregnancy. PubMed, Embase, Scopus, and Web of Science electronic databases were queried to retrieve English-language articles published until April 2020. A reference librarian performed the search. MeSH terms utilized included: “strep*” with “group* b*” or “agalact*,” “gbs,” “vagin*,” “rectum*” or “recto*” or “rectal*,” “perianal*” or “anal*” or “anus*” or “anorect*” or “perineal*” or “perineum*.” We did not initially exclude studies based on design, but ultimately decided to utilize only randomized controlled trials and cohort studies for our analysis. The search was expanded to assess for GBS culturing of specimens obtained from both the vagina and perineum or the vagina and perianal skin, as the perineum and anus have a similar bacterial milieu, thus are comparable structures for swabbing in the process of obtaining samples for culture-based screening for GBS. Additionally, the anus is typically considered to be a perineal structure. We also reviewed the reference lists of all relevant articles to isolate additional pertinent sources.

### Study selection

All titles and abstracts that resulted from the search were independently assessed by two study authors (HCGN and CB). Cases of disagreement were evaluated by a third author (RKE) to determine the appropriateness of inclusion. The PICOS (population, intervention, control/comparator, outcomes, and study design) structure was used to determine the suitability of article inclusion into the study [13]. Articles were excluded for the following reasons: 1) the study did not evaluate a pregnant population, 2) the study did not utilize culture-based GBS screening, 3) the study did not compare vaginal-perianal or vaginal-perineal to vaginal-rectal culture, 4) the study design was not either a randomized controlled trial or a prospective cohort study. After the titles and abstracts were assessed, the same two authors, using the same criteria, assessed the relevant full-text articles independently for inclusion.

The quality of studies was evaluated utilizing the Newcastle–Ottawa Scale [14]. This scale was utilized since all included studies were nonrandomized prospective cohort studies. This tool assessed for quality related to selection, comparability, and outcome of studies. Regarding quality assessment, the tool critically evaluates such factors as cohort representativeness, ascertainment of exposure, comparability of cohorts, assessment of outcome, and adequacy of follow-up. The included studies were deemed to be of high quality, as they were allotted the maximum number of allowable stars for each tested category.

Risk of bias was evaluated using the ROBINS-I tool, given the studies’ nonrandomized nature [15]. This tool assesses for multiple domains of bias, including pre-intervention (such as bias due to confounding and bias in selection of participants into the study), at-intervention (such as bias in intervention classification), and post-intervention (such as bias due to deviation from the intended intervention, bias due to missing data, bias in outcome measurement, and bias in selection of the reported result) domains. The included studies were all determined to have a low risk of bias, as they were rated low-risk across all evaluated domains.

Data extracted from the studies included journal reference, year of publication, corresponding author information, and patient characteristics (age, parity, ethnicity, gestational age). Data on all variables was not required for study inclusion. Additionally, although not included in the meta-analysis, data regarding the pain associated with rectal insertion of the culture swab and the associated patient impression of the experience were collected for review.

Meta-analysis of raw agreement and the Cohen’s kappa statistic were both performed to evaluate the agreement between vaginal-perianal or vaginal-perineal and vaginal-rectal swab tests. Raw agreement, the proportion of overall agreement, was the proportion of cases for which the two tests agreed (either both positive or both negative). Because the raw agreement can be inflated by chance, Cohen’s kappa was also derived. Cohen’s kappa was defined as the proportion of agreement beyond that expected by chance. Landis and Koch criteria were applied to interpret the degree of agreement; ≤ 0: poor, 0.1–0.2: slight, 0.21–0.4: fair, 0.41–0.6: moderate, 0.61–0.8: substantial and 0.81–1: almost perfect [16]. Heterogeneity between studies was assessed by the Cochran’s Q test and quantified by the I^2^ statistic [17]. For both raw agreement and the Cohen’s kappa, due to the absence of heterogeneity, fixed-effects models were used to pool the effect estimates. R software was used, and the *metafor* R package was adopted to perform the meta-analysis [18].

## Results

**Fig. 1:**
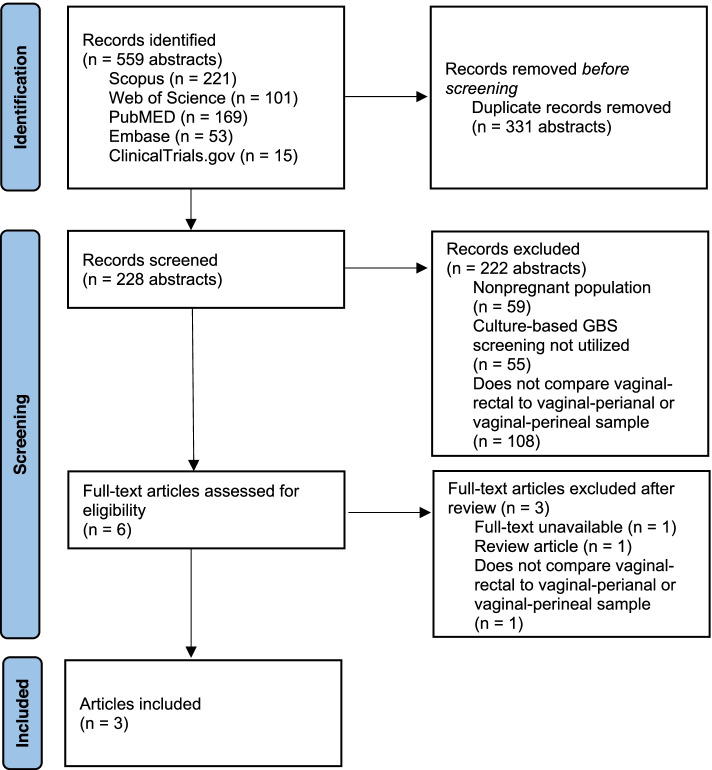
PRISMA flow diagram of retrieved studies in meta-analysis. PRISMA, Preferred Reporting Items for Systematic Reviews and Meta-analyses

Our initial database search returned 559 total records. After exclusions, six articles were deemed relevant for full text review. Following this, three further articles were excluded, as one of the indicated abstracts did not have an available full text article, one was a review article and the last did not compare vaginal-rectal to vaginal-perineal or vaginal-perianal culture as the title and abstract initially suggested. This left a total of three articles for the final analysis (Fig. 1) [11, 12, 19]. These studies were published between 2004 and 2019. The study location was varied, with two of the studies being performed in the United States while the third was performed in Switzerland. No randomized controlled trials were identified, which was expected, as the nature of the question required all study participants to be evaluated with both a vaginal-rectal and a vaginal-perineal or vaginal-perianal culture for GBS. All included studies were prospective cohort studies. The characteristics of included participants and studies are detailed in Tables 1, 2, 3 and 4.

**Table 1:** Baseline characteristics of study participants

Study	Age	Parity	Ethnicity	Gestational Age
Jamie et al. (2004, United States) [11]	25.0 ± 5.5 years	57% parous (reported as dichotomous)	African American 29% Hispanic 8% White 58% Other 4.5%	35.4 ± 3.1 weeks
Trappe et al. (2011, United States) [12]	26.4 ± 5.6 years	71.5% parous (reported as mean 1.5 with standard deviation of 1.5, despite variable being discrete)	African American 24% Hispanic 22.9% Non-Hispanic White 48.7% Asian 1% Other 1.9%	35.9 ± 0.7 weeks
Huang et al. (2019, Switzerland) [19]	32.7 ± 4.6 years	Reported as mean 1 with standard deviation of 1, despite variable being discrete	(Sub)continent of origin • Europe 78.0% • Asia 10.4% • North America 2.0% • Africa 2.0% • Middle East 2.0% • South America 1.6% • Unknown 4.0%	35–37 weeks (mean and variance not reported)

Study references include year of publication and country where the study was undertaken. Data are reported as mean ± standard deviation or proportion of n

**Table 2:** Characteristics of the studies included in the meta-analysis

**Study**	**Study Design**	**Sample Size**	**Comparison groups**	**GBS Detection Rate** **Group 1** **[detected/total tested (%)]**	**GBS Detection Rate** **Group 2** **[detected/total tested (%)]**	**Raw Agreement**	**Cohen’s kappa** **coefficient**
Jamie et al. (2004, United States) [11]	Prospective Cohort	200	1. Vaginal-perianal culture 2. Vaginal-rectal culture	68/200 (34.0%)	67/200 (33.5%)	193/200 (0.97)	0.92
Trappe et al. (2011, United States) [12]	Prospective Cohort	193	1. Vaginal-perianal culture 2. Vaginal-rectal culture	53/193 (27.5%)	56/193 (29.0%)	186/193 (0.96)	0.91
Huang et al. (2019, Switzerland) [19]	Prospective Cohort	250	1. Vaginal-perineal culture, 2. Vaginal-perineal-rectal culture	44/250 (17.6%)	44/250 (17.6%)	242/250 (0.97)	0.89

Study references include year of publication and country where the study was undertaken

**Table 3:** Quality assessment

**Study (year; country)**	**Quality Domains**							
	**Selection**				**Comparability**	**Outcome**		
	**Representativeness of exposed cohort**	**Selection of nonexposed cohort**	**Ascertainment of exposure**	**Demonstration that outcome of interest was not present at start of study**	**Comparability of cohorts on the basis of the design or analysis**	**Assessment of outcome**	**Adequate follow-up length**	**Adequacy of follow up**
Jamie et al. (2004, USA) [11]	*	*	*	*	*	*	*	*
Trappe et al. (2011, USA) [12]	*	*	*	*	*	*	*	*
Huang et al. (2019, Switzerland) [19]	*	*	*	*	*	*	*	*

Quality was assessed using the Newcastle–Ottawa Scale as the included studies were all nonrandomized [14]. The three studies were considered high quality as they were allotted the maximum number of allowable stars for each tested category

**Table 4:** Risk of bias assessment

**Study (year; country)**	**Bias Domains**						
	**Pre-intervention**		**At-intervention**	**Post-intervention**			
	**Bias due to confounding**	**Bias in selection of participants into the study**	**Bias in intervention classification**	**Bias due to deviation from the intended intervention**	**Bias due to missing data**	**Bias in outcome measurement**	**Bias in selection of the reported result**
Jamie et al. (2004, United States) [11]	Low risk	Low risk	Low risk	Low risk	Low risk	Low risk	Low risk
Trappe et al. (2011, United States) [12]	Low risk	Low risk	Low risk	Low risk	Low risk	Low risk	Low risk
Huang et al. (2019, Switzerland) [19]	Low risk	Low risk	Low risk	Low risk	Low risk	Low risk	Low risk

These assessments were performed utilizing the Risk of Bias In Non-randomized Studies – of Interventions (ROBINS-I) tool [15]. The three included studies were considered low risk as they were assessed to be low risk across all domain

The three studies utilized similar specimen collection and culture methods, obtaining samples from each predetermined site on each study patient and utilizing separate swabs for each sample site. The Jamie et al. study used three swabs to collect three different samples: one from the lower third of the vagina, then one from the perianal skin, then one from the rectum. The three swabs were cultured separately, initially in TransVag broth at 35^o^ C for 18–24 h, then further subcultured on 5% sheep’s blood agar plates at 35^o^ C for 18–24 h. The vaginal-perianal specimen was considered positive if either the vaginal or perianal culture, or both, was positive. The vaginal-rectal specimen was considered positive if either the vaginal or rectal culture, or both, was positive. Then vaginal-perianal detection was compared to vaginal-rectal detection to assess for agreement. The Trappe et al. study utilized two swabs to collect two samples: one vaginal-perianal swab, then one vaginal-rectal swab. The two swabs were cultured separately, initially in Lim broth at 35^o^ C for 18–24 h, then the Lim broth was tested for GBS utilizing a GEN-Probe Accuprobe GBS culture identification test. The detection rate of the vaginal-perianal specimen was then compared to the detection rate of the vaginal-rectal specimen to assess for agreement. Finally, the Huang et al. study utilized two swabs to collect two samples: one vaginal-perineal swab, then one rectal swab. The two swabs were cultured separately, initially in Todd-Hewitt broth at 35^o^ C for 24 h, then further subcultured on a selective chromogenic agar medium for GBS (CHROMID Strepto B). The detection rate of the vaginal-perineal specimen was then compared to the detection rate of the combined vaginal-perineal and rectal specimen to assess for agreement.

**Fig. 2:**
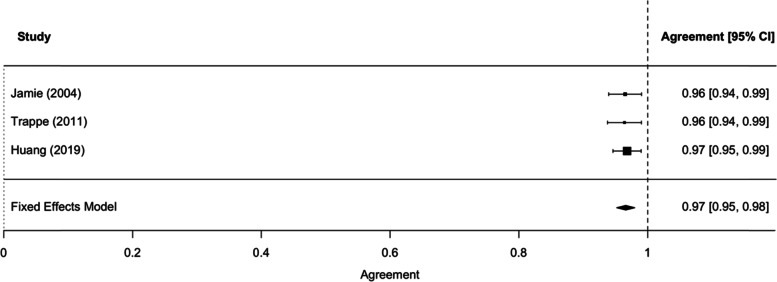
A forest plot of raw agreement estimates and the overall estimate from the fixed-effects model. CI, confidence interval

Within the three included studies, GBS was detected utilizing both methods (vaginal-perianal or vaginal-perineal culture and vaginal-rectal culture) at a rate of 17.6–34.0%, which is consistent with the anticipated frequency reported in earlier publications describing GBS detection rate with serial culture in selective broth media and then on blood agar plates [3, 4]. For raw agreement, the test for heterogeneity showed that there was no obvious heterogeneity among studies (*Q* = 0.06, *df* = 2, *p* = 0.97, *I*^*2*^ = 0%). The funnel plot did not show evidence of publication bias. Based on the fixed-effects model, the pooled estimate of the raw agreement was 0.97 (95% CI: 0.95–0.98)—see Fig. 2.

**Fig. 3:**
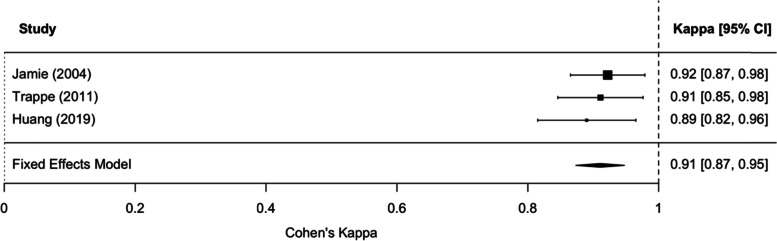
A forest plot of Cohen’s kappa estimates and the overall estimate from the fixed-effects model. CI, confidence interval

As described in the methods, raw agreement comparisons can be inflated by chance. Therefore, Cohen’s kappa coefficient was also evaluated. For Cohen’s kappa coefficient, the test for heterogeneity was not significant, indicating there was not considerable heterogeneity among studies (*Q* = 0.4449, *df* = 2, *p* = 0.8005, *I*^*2*^ = 0%). From the funnel plot, we did not see any evidence of publication bias. Based on the fixed-effects model, the pooled estimate of the Cohen’s kappa coefficient was 0.91 (95% CI: 0.87–0.95), which indicated an almost perfect agreement between the two clinical tests according to the Landis and Koch criteria—see Fig. 3.

Landis and Koch criteria for interpreting the degree of agreement reflected by Cohen’s kappa coefficient are as follows: ≤ 0: poor; 0.1–0.2: slight; 0.21–0.4: fair; 0.41–0.6: moderate; 0.61–0.8: substantial; and 0.81–1: almost perfect.

As can be seen in Table 1, most patients in all three studies were parous, and all patients were in the third trimester of pregnancy. Patients in the Jamie et al. and the Trappe et al. studies were younger (25.0 ± 5.5 years and 26.4 ± 5.6 years, respectively) than those in the study by Huang et al. (32.7 ± 4.6 years). Ethnicity in the two studies completed in the United States were similarly allocated, with a majority of participants being white or African American. The Huang et al. study, completed in Switzerland, had a much more geographically diverse population, with participants originating from at least six regions or continents around the world, despite the majority (78%) originating from Europe.

Two of the three studies assessed the patient’s experience and perception of discomfort following insertion of the rectal swab. Participants in the Huang et al. study were asked, immediately after collection of culture specimens, to mark on a visual analog scale the level of stress, discomfort, or pain, ranging from 0 to 10 (with 0 representing no stress, discomfort, or pain, while 9.5–10 was denoted as maximal stress, discomfort, or pain). The majority of participants tolerated the procedure well, but of the 246 women who completed the scale for discomfort, greater than 70 participants rated the discomfort as either moderate or maximal. In the Trappe et al. study, participants completed a short questionnaire in which they rated the pain with both collection methods on a 0 to 10 scale (with 0 representing no pain and 10 representing extremely severe pain). They then compared the discomfort between the two tests. The pain rating was noted to be, on average, 2.2 points higher on the 0–10 scale with vaginal-rectal compared to vaginal-perianal swabbing (mean ± standard deviation 3.4 ± 2.9 compared to 1.2 ± 1.7). Twenty-five percent of participants rated their pain as six or higher with vaginal-rectal swabbing. Further, 68.4% rated the pain as worse with the vaginal-rectal swabbing than vaginal-perianal swabbing (132 of the 193 respondents). Patient experience was not assessed in the study by Jamie et al.

## Discussion

Routine screening for GBS and the use of intrapartum antibiotic prophylaxis has reduced the prevalence of early-onset neonatal sepsis, pneumonia, and meningitis due to GBS infection in the neonate. As such, antenatal culture-based screening of the gravid patient remains an important strategy to identify GBS carriers prior to delivery. The most updated guidelines regarding sampling techniques continue to recommend that a culture be performed utilizing a specimen collected with a single swab of the lower vagina (near the introitus) and then of the rectum (through the anal sphincter) [6]. In this systematic review and meta-analysis, we have demonstrated that rectal sampling is unnecessary and associated with more discomfort than sampling the perineum or perianal skin.

In order to “maximize the likelihood of GBS recovery”, the guideline [6] cites studies that compared combined vaginal and rectal sampling to either the cervix alone or the vagina alone [20–22]. The comparison of combined vaginal and rectal sampling with combined vaginal and perianal or perineal sampling is just ignored by the guideline [6]. None of the studies cited by the guideline assessed the utility of sampling sites other than the cervix, vagina or rectum. It is interesting that multiple other countries advocate for sampling only from the vagina [23] or from the vagina and perianal area [24, 25] for assessing for GBS colonization.

This systematic review and meta-analysis compiled data from three identified studies that evaluated for GBS colonization in 643 pregnant third trimester women utilizing vaginal-perianal or vaginal-perineal culture compared to vaginal-rectal culture [11, 12, 19]. Individually, all three of these studies support the use of vaginal-perianal or, more broadly, vaginal-perineal sampling (as the perineum contains the anus) instead of vaginal-rectal sampling which may serve as a valid and more comfortable alternative to vaginal-rectal sampling. These studies individually have not led to a change in the recommended mode of specimen collection by organizations such as the CDC, ACOG, or the APA, likely due to the relatively small number of women tested in each single site study, despite appropriate power analyses being completed in each study. We think that, collectively, these data should prompt a change in the guideline since the pooled data show “almost perfect” agreement [26] between vaginal-perineal and vaginal-rectal sampling.

This systematic review and meta-analysis has multiple strengths. The study design, including strategies for searches and selection of studies for inclusion, were predetermined in a review protocol. This review systematically identified all relevant publications assessing the detection rate and agreement of vaginal-perianal or vaginal-perineal and vaginal-rectal cultures in the detection of GBS in pregnant women. The included studies were all found to have a low risk of bias and were noted to be of high quality. As there was no obvious heterogeneity, a meta-analysis was able to be performed, which validated findings from the individual studies, but in the setting of multiple sites, both in the US and internationally, within a larger patient cohort. Finally, the statistical method utilized determined that agreement between methods was unlikely conflated by chance.

Regarding potential limitations, the authors of Huang et al. commented as a potential limitation of their study, that as the Swiss Society of Gynecologists and Obstetricians (SGGG) guidance has led to the broader adoption of vaginal-perineal swabbing to avoid subjecting the patient to the discomfort associated with rectal swabbing, many women who were approached to participate in the study elected not to do so due to fear of pain associated with rectal swabbing. As such, the authors speculated that this may have led to participation by women who were less pain-sensitive or stressed, so that article may actually underestimate the amount of pain associated with rectal sample collection for GBS culture.

Another potential limitation was in the methodological differences utilized for the specimen collection, culturing and subculturing of the swabs in the three included studies. All included studies adhered to guidance from the CDC available at the time the studies were completed regarding the collection and processing of clinical specimens for culture of GBS. The use of the same swab or different swabs for specimen collection across multiple sites was recommended by the CDC, and, as was the case in the included studies, the results from the culturing is ultimately integrated to determine whether or not GBS is present. Similarly, while culturing and subculturing processes for GBS varied across included studies, the GBS detection rates for included studies are high. Despite this, it is possible that the variance in technique may have led to differing rates of GBS detections between the studies. [2, 27].

Others have also evaluated patient discomfort with various sites of collection of swab specimens for GBS screening cultures. In a prospective cohort study, Orafu et al. compared perianal, vaginoperianal, and anorectal sampling for GBS culture in pregnant women [10]. Following specimen collection, the clinician obtaining the specimen asked the subject to describe her perception of pain none, mild, moderate, or severe. Sixty-eight percent of subjects described their pain as mild to moderate, and five percent described their pain as severe. The study authors’ statistical analysis using the Pearson χ^2^ test found unequally distributed pain scores (χ^2^ = 44.882, *P* = 9.80 × 10^−10^). The study did not ultimately qualify for this meta-analysis despite finding perianal sampling to be equivalent to anorectal sampling for GBS detection since these authors did not compare combined vaginal-perianal sampling to combined vaginal-anorectal sampling.

In summary, this systematic review and meta-analysis evaluating the agreement between vaginal-perianal or vaginal-perineal and vaginal-rectal cultures for screening for maternal anogenital GBS colonization in third trimester pregnant patients provides evidence that should lead clinicians to a practice change regarding specimen collection. This evaluation’s narrow focus allowed for a critical analysis of a broad body of literature that supports the findings put forward previously in the multiple smaller studies. The results of both high levels of raw agreement and a favorable Cohen’s kappa coefficient within the larger sample size identified within the systematic review is reassuring. Further, eliminating rectal specimen collection would lead to a clinically-relevant decrease in patient discomfort.

## Data Availability

All data generated or analysed during this study are included in this published article.
